# Synergy in symbiosis

**DOI:** 10.7554/eLife.85565

**Published:** 2023-02-03

**Authors:** Aileen Berasategui, Hassan Salem

**Affiliations:** 1 Mutualisms Research Group, Max Planck Institute for Biology Tübingen Germany

**Keywords:** *Apis mellifera*, microbiome, metabolism, Bifidobacterium, amygdalin, Other

## Abstract

Honeybees rely on their microbial gut symbionts to overcome a potent toxin found in pollen and nectar.

**Related research article** Motta EVS, Gage A, Smith TE, Blake KJ, Kwong WK, Riddington IM, Moran N. 2022. Host-microbiome metabolism of a plant toxin in bees. *eLife* **11**:e82595. doi: 10.7554/eLife.82595.

Plants deploy a remarkable range of mechanical structures (such as thorns) and chemical weapons (such as toxins) to protect themselves against herbivores. Secondary metabolites from plants can harm herbivores by disrupting the transport of nutrients or the production of hormones. However, herbivores fight back by detoxifying, isolating or deactivating these metabolites (Mithöfer and Boland, 2012).

Gut microbes in herbivores are also involved in breaking down toxins into less dangerous chemicals (Dearing et al., 2022). Now, in eLife, Nancy Moran and colleagues at the University of Texas at Austin and the Gulbenkian Institute in Portugal – including Eric Motta as first author – report how symbiotic microbes can upgrade the detoxifying capacity of the Western honeybee against amygdalin, a widespread plant toxin (Motta et al., 2022).

Amygdalin is a cyanogenic compound that is found in the nectar and pollen of many of the plant species that bees frequent, including almond, apple and cherry trees. Although bees can fully degrade amygdalin, the mechanism behind this metabolism remained elusive.

Herbivores typically encode specific enzymes that help to break down plant toxins (Erb and Reymond, 2019). However, compared to other insects, bees encode relatively few of these genes, suggesting that the gut microbiome may be involved in degrading plant toxins, including amygdalin.

To test this hypothesis, Motta et al. compared the effects of amygdalin consumption in bees with and without their native gut microbes. They found that honeybees without their microbes can only partially degrade amygdalin into an intermediate, called prunasin. In honeybees harbouring gut microbes, on the other hand, amygdalin is fully broken down into hydrogen cyanide and other derivatives without apparent harmful effects on the bees (Figure 1).

**Figure 1.**
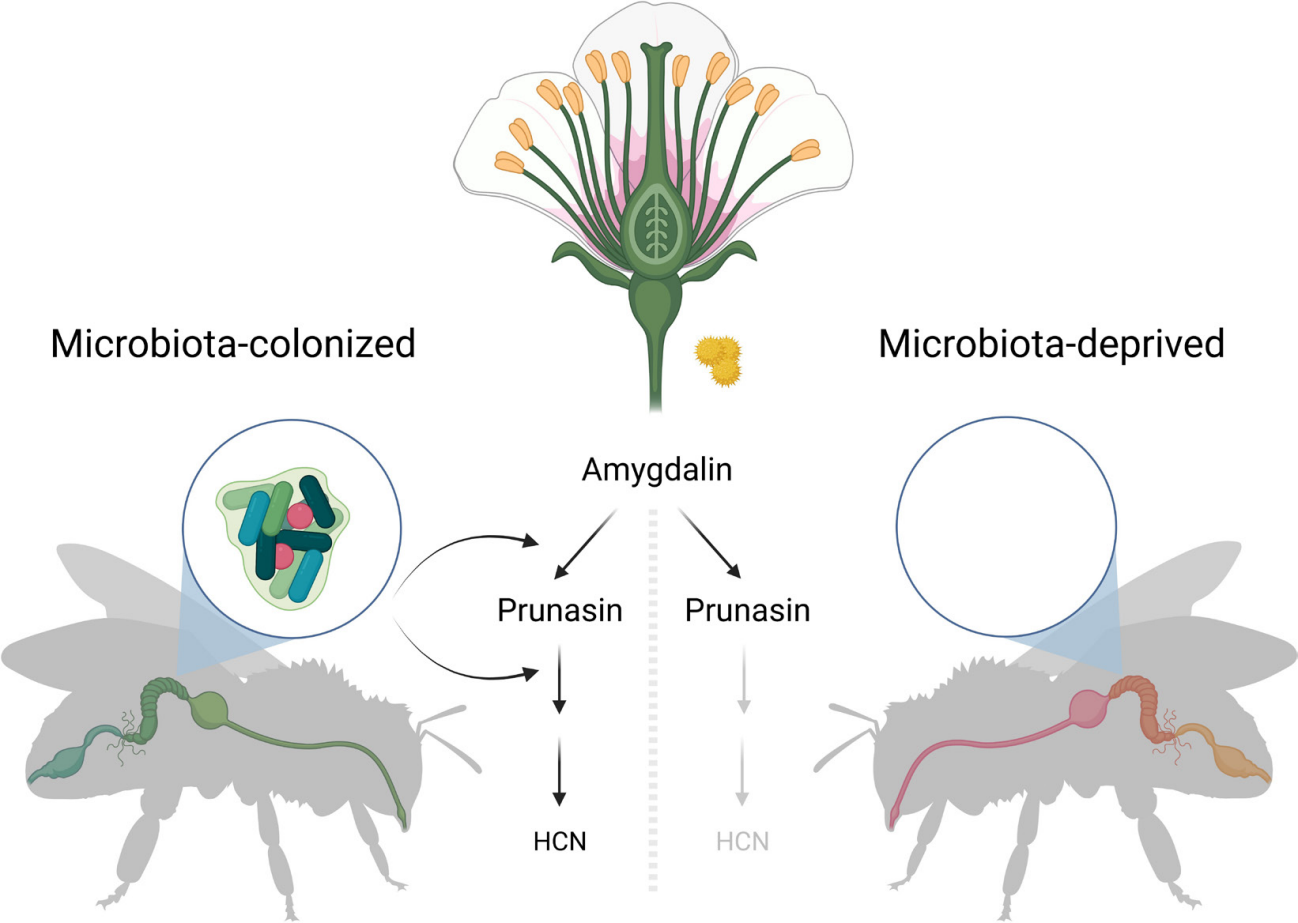
Metabolising plant toxins in the honeybee gut. Amygdalin is a toxin found in the nectar and pollen of several plants, and it can be harmful if ingested in large quantities. Motta et al. found that bees with gut bacteria (left) were able to fully degrade amygdalin into hydrogen cyanide (HCN) and other metabolites. However, bees without gut bacteria (right) were only able to degrade the chemical to an intermediate compound, called prunasin. This confirmed that enzymes secreted by gut bacteria are essential to fully degrade amygdalin.

Image created with BioRender.com.

Motta et al. then examined the different types of gut bacteria and their ability to cope with varying concentrations of amygdalin, and quantified the metabolic by-products. This revealed that only a few types of bacteria, including a strain of the genus *Bifidobacterium*, can break down amygdalin. Introducing this bacterial strain into honeybees without gut bacteria enabled them to fully degrade amygdalin, mirroring what happens in bees that harbour a native community of microbes.

Motta et al. then set out to identify the enzymes secreted by *Bifidobacterium* that are responsible for the break down. When grown in cultures containing amygdalin, the bacterium upregulated the expression of a gene encoding for glycoside hydrolase (GH) family 3, which belongs to a class of enzymes that is known to degrade amygdalin. Expressing the gene encoding GH3 in *E. coli*, which cannot normally degrade amygdalin, enabled it to break down the metabolite. This confirms the importance of GH3 for the metabolism of amygdalin.

Microbial symbionts are increasingly recognized for their role in reducing the toxicity of plant metabolites in insects. Local environments with a high abundance of toxins may exert a strong selection on microbes in these locations to degrade plant metabolites (Hammer and Bowers, 2015). Herbivores may leverage this metabolic diversity by acquiring new symbionts directly from the environment, or through the exchange of genetic material (e.g., horizontal gene transfer) between gut bacteria (Berasategui et al., 2017; Ceja-Navarro et al., 2015).

Gut symbionts in bees modulate a remarkable range of functions in their hosts, from fermenting complex sugars to fighting off parasites (Kwong et al., 2017; Kwong and Moran, 2016). Motta et al. show that these froles extend to metabolizing plant toxins. Characterizing amygdalin metabolism across different bee species would help clarify if the ability to fully degrade the toxin is a fixed feature of the gut microbiome, or if it is shaped by the environment and the distribution of amygdalin-producing plants. Ultimately, a better understanding of how hosts and gut symbionts interact to degrade toxins, may shed light on the effect of various plant metabolites on bee health, and the different strategies to counteract them.
